# Measuring colorectal cancer care quality for the publicly insured in New York State

**DOI:** 10.1002/cam4.30

**Published:** 2012-11-22

**Authors:** Amber H Sinclair, Maria J Schymura, Francis P Boscoe, Rachel L Yung, Kun Chen, Patrick Roohan, Eric Tai, Deborah Schrag

**Affiliations:** 1 New York State Department of Health, Cancer RegistryAlbany, NY; 2 Department of Medical Oncology, Center for Outcomes and Policy Research, Dana-Farber Cancer InstituteBoston, MA; 3 New York State Department of Health, Office of Health Insurance ProgramsAlbany, NY; 4 Centers for Disease Control and PreventionAtlanta, GA

**Keywords:** Colorectal cancer, guideline adherence, Medicaid, Medicare, quality of care

## Abstract

The extent to which concordance with colorectal cancer treatment quality metrics varies by patient characteristics in the publicly insured is not well understood. Our objective was to evaluate the quality of colorectal cancer care for publicly insured residents of New York State (NYS). NYS cancer registry data were linked to Medicaid and Medicare claims and hospital discharge data. We identified colorectal cancer cases diagnosed from 2004 through 2006 and evaluated three treatment quality measures: adjuvant chemotherapy within 4 months of diagnosis for American Joint Cancer Committee (AJCC) stage III colon cancer, adjuvant radiation within 6 months of diagnosis for AJCC stage IIB or III rectal cancer, and adjuvant chemotherapy within 9 months of diagnosis for AJCC stage II–III rectal cancer. Concordance with guidelines was evaluated separately for Medicaid-enrollees under age 65 years and Medicare-enrollees aged 65–79 years. For adjuvant chemotherapy for colon cancer, 79.4% (274/345) of the Medicaid cohort and 71.8% (585/815) of the Medicare cohort were guideline concordant. For adjuvant radiation for rectal cancer, 72.3% (125/173) of the Medicaid cohort and 66.9% (206/308) of the Medicare cohort were concordant. For adjuvant chemotherapy for rectal cancer, 89.5% (238/266) of the Medicaid cohort and 76.0% (392/516) of the Medicare cohort were concordant. Younger age was associated with higher adjusted odds of concordance for all three measures in the Medicare cohort. Racial differences were not evident in either cohort. There is room for improvement in concordance with accepted metrics of cancer care quality. Feedback about performance may assist in targeting efforts to improve care.

## Introduction

Colorectal cancer is the second most common cancer and the second most common cause of cancer death among cancers affecting men and women in the United States [1]. In New York State (NYS), approximately 10,000 new cases of colorectal cancer are diagnosed and about 4000 men and women die from the disease each year [2]. Appropriate colorectal cancer treatment, especially in the early stages, can increase the survival rate and reduce the chances the cancer will reoccur [3].

To develop a standard set of cancer care quality metrics, in 2007, the National Comprehensive Cancer Network (NCCN) and the American Society of Clinical Oncology (ASCO) collaborated to identify three care quality measures for the treatment of colorectal cancer: (1) adjuvant chemotherapy for patients with American Joint Cancer Committee (AJCC) stage III colon cancer within 4 months (120 days) of diagnosis, (2) adjuvant radiation for clinical or pathologic AJCC stage IIB [T4 N0 M0] or III rectal cancer within 6 months (180 days) of diagnosis, and (3) adjuvant chemotherapy for AJCC stage II–III rectal cancer within 9 months (270 days) of diagnosis [4, 5]. In addition to NCCN and ASCO, the Commission on Cancer (CoC) collaborated and agreed upon the specifications of the measures of radiation for rectal cancer and adjuvant chemotherapy for colon cancer, and the National Quality Forum also endorsed the measure of adjuvant chemotherapy for colon cancer [4].

The NCCN/ASCO measures include detailed specifications, which allow for comparison of results of standardized measures across studies and can be assessed using cancer registry and claims data. The guideline developers evaluated performance at eight NCCN centers [6]. At these hospitals which have large specialty practices, mean concordance with guidelines was 90% for receipt of adjuvant chemotherapy within 4 months of diagnosis for stage III colon cancer, 93% for receipt of radiation therapy within 6 months of diagnosis for clinical or pathologic AJCC T4N0M0 or stage III rectal cancer, and 81% for receipt of adjuvant chemotherapy within 9 months of diagnosis of stage II–III rectal cancer.

The purpose of our study was to evaluate concordance with quality metrics for Medicaid and Medicare insured colorectal cancer patients in NYS using tumor registry data linked with administrative claims and hospital discharge and ambulatory surgery records. This approach followed the Institute of Medicine's recommendation that central cancer registries be linked to claims and hospital discharge files to provide state or national data on the quality of cancer care [7], and built on the Institute of Medicine's Committee on Assessing Improvements in Cancer Care in Georgia report which translated care quality guidelines into specific metrics that could be applied at the state level [8]. Although other studies have evaluated concordance with guideline therapy for colorectal cancer [6, 9–19], what is unique about this study is its inclusion of the Medicaid population with Medicaid claims data. Quality of care studies indicate that care is inferior for vulnerable populations, including racial and ethnic minorities [20]. The important contribution of this work is that we focus on a vulnerable population by virtue of their receiving publicly funded Medicaid insurance.

## Methods

### Data

We identified colorectal cancer cases diagnosed in NYS residents from 2004 through 2006 in the NYS Cancer Registry (NYSCR). The cancer cases were linked to Medicaid and Medicare claims data, and to the NY Statewide Planning and Research Cooperative System (SPARCS) hospital discharge and ambulatory surgery data. Medicaid and SPARCS data were obtained through internal NYS Department of Health (NYSDOH) sources, whereas Medicare data were obtained from the Research Data Assistance Center at the University of Minnesota. Data sources were linked using a combination of identifying information, which varied by data source. Details on the linkage of the Medicaid and NYSCR data sets have been previously described [21]. Institutional Review Board (IRB) approval was obtained from both the NYSDOH and the Dana Farber Cancer Institute.

Covariate data obtained from the NYSCR included gender, age, race/ethnicity, and marital status. We combined race and ethnicity to create four groups for the colon cancer measure: Hispanic, non-Hispanic white, non-Hispanic black, and non-Hispanic other. The numbers by race were smaller for the two rectal cancer measures, so for these we combined race and ethnicity as follows: Hispanic, non white non-Hispanic, and white non-Hispanic. We used U.S. Census data to obtain a measure of median household income from census tracts of patient home addresses. For geographic region, we combined residential county codes into regions representing New York City (NYC), the suburbs of NYC, upstate urban, and upstate rural areas. Hospital size was determined by the number of beds of the hospital of primary surgery as recorded in the NYSCR. Bed size data were obtained from the NYS hospital profile data in the Health Facilities Master File and categorized into small (<100 beds), medium (100–400 beds), and large facilities (>400 beds). The comorbidity measure was ascertained using the Charlson–Deyo–Klabunde comorbidity index [22–24] applied to Medicaid and Medicare claims for the 1 year prior to diagnosis.

### Quality measures

All three measures had the following inclusion criteria: age <80 years at time of diagnosis, alive throughout the time frame of the measure, known or assumed to be first or only cancer diagnosis, epithelial malignancy only, received surgery for the primary site, and continuously enrolled in Medicaid or Medicare for the time period relevant to the measure. The continuous enrollment specification allowed for 1 month of discontinuous enrollment during the measure time period. For the quality measure denominator specification of surgery of the primary site, we included evidence of surgery from the registry or any of the linked sources.

The outcome of interest was receipt of radiation or chemotherapy within the specified time period and after the surgery date for each of the three measures. If treatment was identified in any of the combined data sources during the specified time period for a given measure, it was recorded as present. In the cancer registry, treatment was identified from radiation and chemotherapy treatment fields. For Medicaid, Medicare, and SPARCS data, receipt of treatment was identified using International Classification of Diseases (ICD-9) diagnosis and procedure codes, Current Procedural Terminology (CPT) codes, and Healthcare Common Procedure Coding System (HCPCS) codes. For the Medicaid claims and Medicare Part D claims (only for 2006–2007), chemotherapy was also identified using National Drug Codes. A list of radiation and chemotherapy treatment codes is available from the authors by request.

Enrollment in public insurance was determined from Medicaid and Medicare monthly enrollment records. Only those patients who were enrolled in a Medicare fee-for-service plan and in Parts A and B during the measure time period were included in the Medicare cohort. All the Medicaid enrollees were included since NYS Medicaid tracks monthly enrollment and maintains records of claims and encounter files for all medical services provided to plan members. Medicaid claims files capture services for fee-for-service enrollees and encounter files provide similar detailed information as claims, but are generated for managed care enrollees. In our analysis, patients who were dually enrolled in both Medicaid and Medicare and under age 65 years were included in the Medicaid cohort. Patients who were dually enrolled and ages 65–79 years were included in the Medicare cohort.

### Analysis

We ran parallel analysis for each measure for the Medicaid cohort and the Medicare cohort. We assessed the proportion of patients meeting each of the three quality measures by the characteristics given in Table 1. We used logistic regression modeling to estimate the crude and adjusted odds ratios with 95% confidence intervals. The adjusted multivariate models included all of the covariates. Missing values were excluded from the analysis. The number of cases excluded from the multivariate models ranged from 16 (of 345) for the measure of chemotherapy within 4 months of diagnosis for stage III colon cancer in the Medicaid population to 74 (of 516) for the measure of chemotherapy within 9 months of diagnosis for stage II–III rectal cancer in the Medicare population. Most of the cases were deleted due to missing values for marital status, the number of positive lymph nodes, and/or hospital bed size information. In general, the cases that were deleted were similar on covariate distributions to the cases that were included in the models, with one consistent exception for all three measures for Medicaid and Medicare: the deleted cases were more often from rural geographic areas.

**Table 1:** Descriptive statistics for colon and rectal cancer quality measure denominator populations, diagnosed 2004–2006

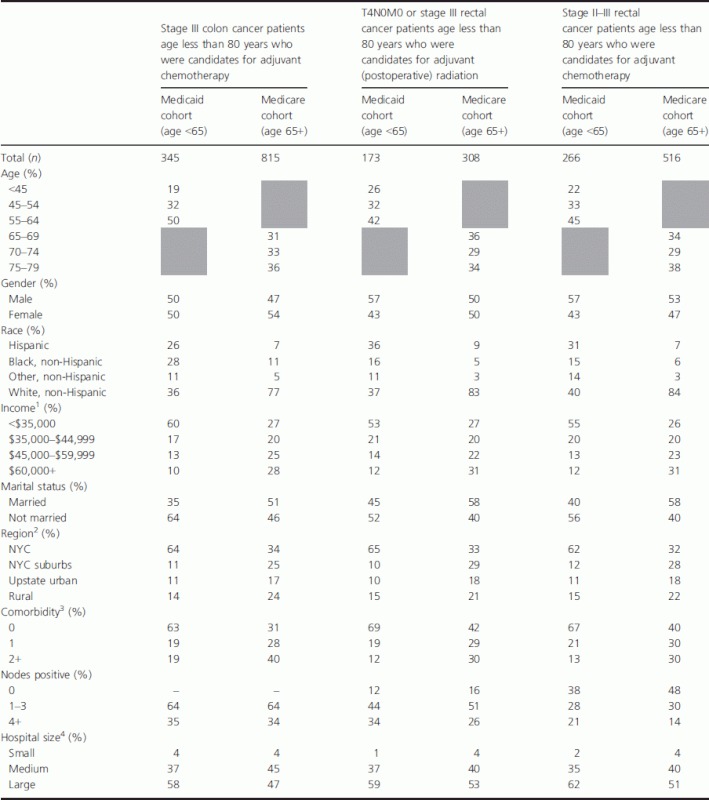

Percentages may not add to 100% due to missing values and rounding.

^1^Median household income based on census tract of patient home address.

^2^Region based on grouping of residential county codes.

^3^Comorbidity was ascertained using the Charlson–Deyo–Klabunde Index applied to Medicaid and Medicare claims.

^4^Hospital size based on number of beds.

We also conducted a sensitivity analysis that extended the time intervals for each quality measure to determine delayed compliance with each measure: 4 months was extended to 6 months, 6 months was extended to 9 months, and 9 months was extended to 12 months.

## Results

The distribution of patient characteristics in each cohort for each measure is shown in Table 1. Compared with Medicare, the Medicaid cohort for each measure had lower income, a higher proportion of minority patients, fewer married patients, more comorbidity, a higher proportion with lymph node positive rectal cancer, and more often had primary surgery in larger hospitals.

For receipt of adjuvant chemotherapy within 4 months of diagnosis for AJCC stage III colon cancer patients, there was 79.4% concordance in the Medicaid cohort and 71.8% concordance in the Medicare cohort (Table 2). None of the variables were significant in the adjusted analyses for the Medicaid cohort for this measure. For the Medicare cohort, the adjusted odds of receiving guideline recommended treatment was significantly higher for younger age, and was significantly lower for men compared to women and for those with two or more comorbidities compared to none.

**Table 2:** Adjuvant chemotherapy is administered within 4 months (120 days) of diagnosis for patients under the age of 80 years with AJCC stage III (lymph node positive) colon cancer

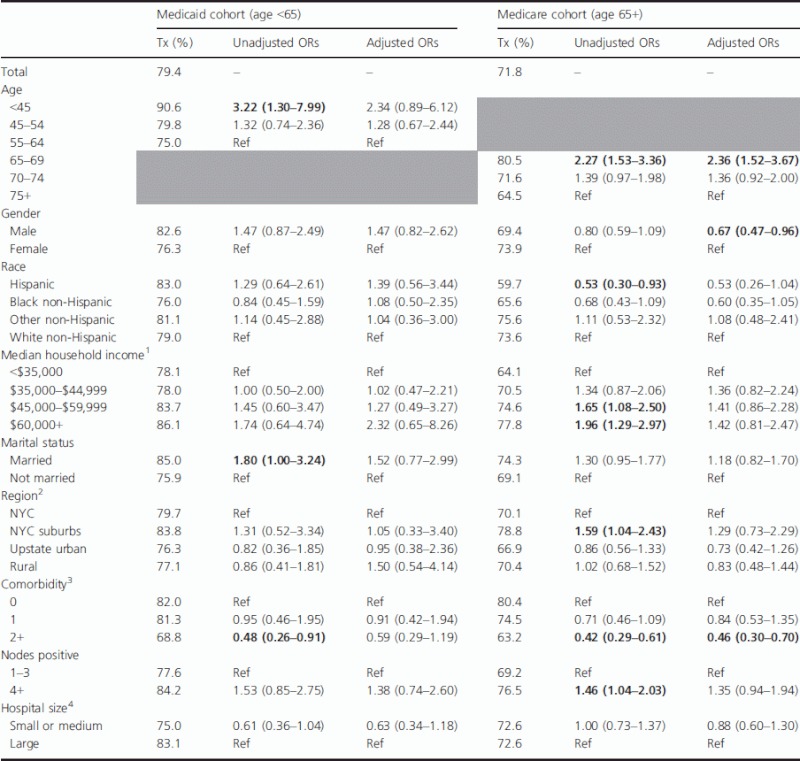

Boldface signifies significant values.

^1^Median household income based on census tract of patient home address.

^2^Region based on grouping of residential county codes.

^3^Comorbidity was ascertained using the Charlson–Deyo–Klabunde Index applied to Medicaid and Medicare claims.

^4^Hospital size based on number of beds.

For receipt of adjuvant radiation therapy within 6 months of diagnosis for clinical or pathologic AJCC stage IIB or III rectal cancer patients, there was 72.3% concordance in the Medicaid cohort and 66.9% concordance in the Medicare cohort (Table 3). None of the variables examined were significant for the Medicaid cohort for this measure. For the Medicare cohort, the adjusted odds of receiving guideline recommended treatment were significantly higher for younger age. The odds of receiving treatment were also significantly higher for those with either 0 positive lymph nodes or 4+ positive lymph nodes compared with 1–3 positive lymph nodes.

**Table 3:** Radiation therapy is administered within 6 months (180 days) of diagnosis for patients under the age of 80 years with clinical or pathologic AJCC T4N0M0 or stage III receiving surgical resection for rectal cancer (postoperatively)

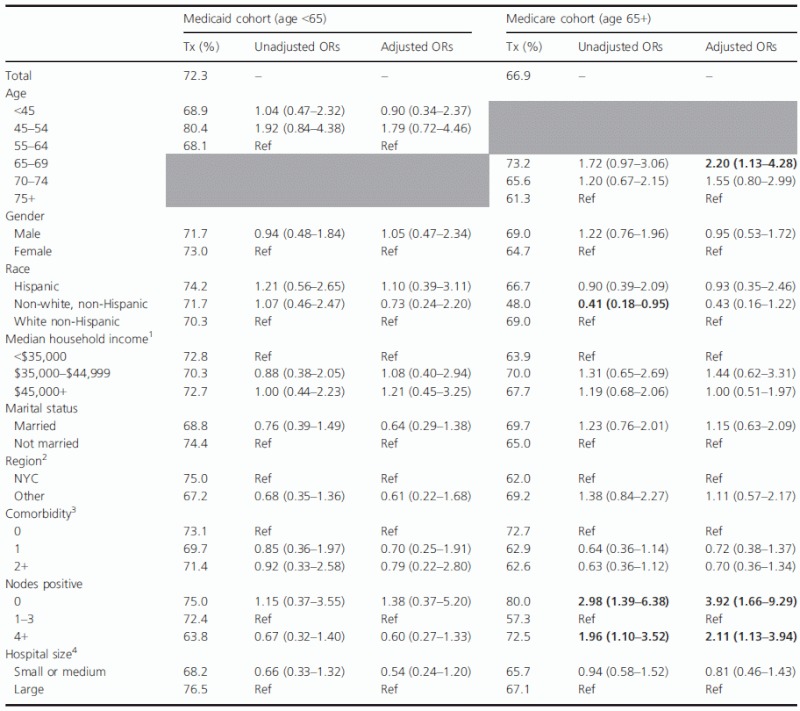

Boldface signifies significant values.

^1^Median household income based on census tract of patient home address.

^2^Region based on grouping of residential county codes.

^3^Comorbidity was ascertained using the Charlson–Deyo–Klabunde Index applied to Medicaid and Medicare claims.

^4^Hospital size based on number of beds.

For receipt of adjuvant chemotherapy within 9 months of diagnosis for AJCC stage II or III rectal cancer patients, there was 89.5% concordance in the Medicaid cohort and 76.0% concordance in the Medicare cohort (Table 4). In adjusted analyses for the Medicaid cohort for this measure, the odds of receiving guideline recommended treatment were significantly lower for NYC suburbs compared to NYC and were significantly higher for small or medium hospital size compared to large hospital size. Just as in the other two measures, in the Medicare cohort the adjusted odds of receiving treatment were significantly higher for ages 65–69 years. Adjusted odds were also significantly higher for ages 70–74 years compared to 75+. The odds were significantly lower for 0 positive lymph nodes compared to 1–3 positive lymph nodes.

**Table 4:** Postoperative adjuvant chemotherapy is administered within 9 months (270 days) of diagnosis for patients under the age of 80 years with AJCC stage II or stage III rectal cancer

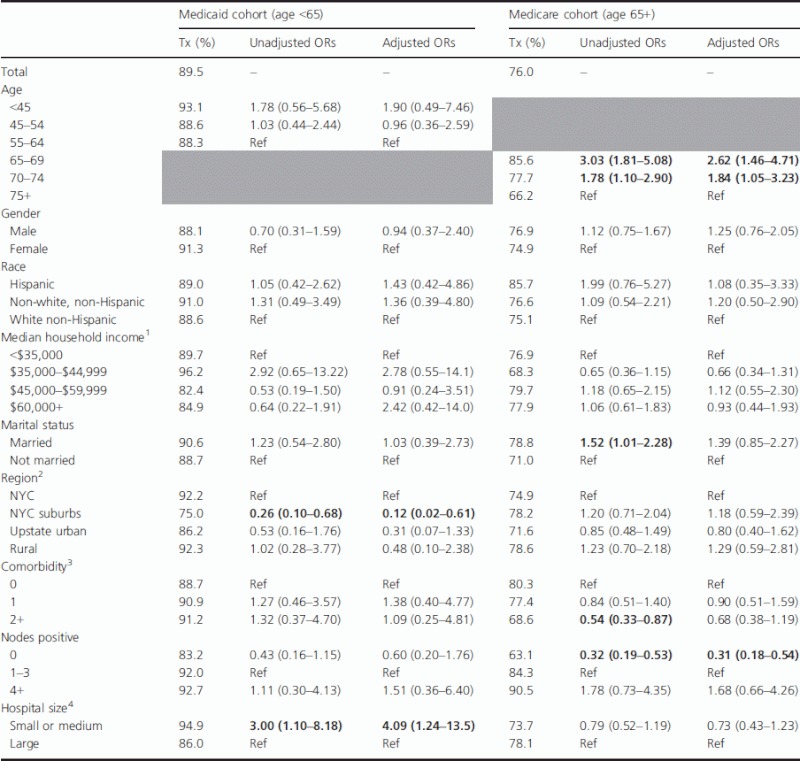

Boldface signifies significant values.

^1^Median household income based on census tract of patient home address.

^2^Region based on grouping of residential county codes.

^3^Comorbidity was ascertained using the Charlson–Deyo–Klabunde Index applied to Medicaid and Medicare claims.

^4^Hospital size based on number of beds.

The percent of patients who had adjuvant chemotherapy for colon cancer increased from 79.4% to 87.9% for Medicaid and from 71.8% to 78.7% for Medicare when extending the time period from 4 to 6 months. The percent of patients who had radiation therapy postsurgery for rectal cancer increased from 72.3% to 77.4% for Medicaid and from 66.9% to 68.4% for Medicare when extending the time period from 6 to 9 months. Finally, the percent of patients who had adjuvant chemotherapy for rectal cancer increased from 89.5% to 90.2% for Medicaid and from 76.0% to 77.8% for Medicare when extending the time period from 9 to 12 months.

## Discussion

We evaluated concordance with nationally recognized colorectal cancer quality measures for publicly insured residents in NYS. Although the majority of patients receive care that is guideline concordant, there is nevertheless room for improvement. Specifically, 21% of Medicaid-enrolled and 28% of Medicare-enrolled stage III colon cancer patients did not receive adjuvant chemotherapy within 4 months, 28% of Medicaid-enrolled and 33% of Medicare-enrolled stage IIB or III rectal cancer patients did not receive adjuvant radiation within 6 months, and 10% of Medicaid-enrolled and 24% of Medicare-enrolled stage II or III rectal cancer patients did not receive adjuvant chemotherapy within 9 months.

An NCCN-based study has also evaluated concordance to NCCN/ASCO guidelines [6]. We had lower rates of radiation for rectal cancer and chemotherapy for colon cancer, but similar rates of chemotherapy for rectal cancer. It is not unexpected that guideline concordance was lower in our study than those of the eight NCCN cancer care centers, given that our study is population-based, capturing general community practice as well as care provided at specialized cancer centers.

The most consistent finding in our analysis was that older patients were independently and significantly less likely to receive treatment for all three measures for the Medicare cohort. Although not statistically significant, we also found higher odds of treatment for younger age groups compared to the oldest age group (55–64) in the Medicaid cohort. This finding suggests that despite the evidence-based guideline recommendation of treatment for all patients under age 80 years, clinical practice has not followed suit. Our study adds to the literature demonstrating under-treatment of elderly patients with cancer [9–16]. For example, a study using California cancer registry data found that use of adjuvant therapy for colorectal cancer was significantly lower for older patients [14]. Another study that used Michigan Tumor Registry data found that older patients were less likely to initiate adjuvant chemotherapy for colon cancer [15]. Similarly, a study by Cress et al. based on the multi-state CDC-NPCR Patterns of Care Study using 1997 data looked at adjuvant chemotherapy for patients with stage III colon cancer, finding lower adjuvant chemotherapy use for older age patients [16].

It is notable that we did not find evidence of significant racial disparities within either the Medicaid or Medicare populations, despite a relatively large percentage of minorities particularly in the Medicaid cohort. The lack of a racial disparity in our findings is inconsistent with some studies that have looked at race differences in colorectal cancer treatment [11, 12, 14, 17], yet consistent with others [13, 16, 18, 19]. One explanation for why we did not find a racial difference in the Medicaid population could be that racial differences in cancer care are often mediated by socioeconomic status [25], and in the Medicaid program all patients are poor. For our Medicare population, we have small numbers of minorities resulting in less power to detect a statistical difference by race/ethnicity.

There are some limitations to this analysis. First is the lack of information regarding patient and provider interactions and decision making. It is possible that there are patients for whom treatment may not be appropriate, such as a limited life expectancy given serious other unmeasured comorbidity or strong patient preference. The NCCN study assessed reasons for non-adherence, and found that lack of complete documentation, patient refusal, delayed treatment initiation, and lack of consensus on necessity of treatment were reasons for non-adherence [6]. Second, there are challenges inherent in registry and claims data. Each of the data sources is subject to errors in recording of information. However, the combination of data sources improves the accuracy of treatment information. Third, small numbers of racial populations limited our ability to look at findings by race other than white and non-white categories for the two rectal cancer measures. Finally, as noted in the Methods section, the analysis of the Medicare population includes only those patients who are enrolled in a fee-for-service plan since Medicare claims data for HMO plans are not available.

In summary, although the majority of patients receive care that is guideline recommended treatment, there is nevertheless room for improvement in concordance to accepted metrics of cancer care quality. Although treatment is recommended in patients up to age 80 years, older patients were less likely to receive treatment than younger patients. We did not find disparities by race/ethnicity. Because our study was conducted at the state health department, which administers the Medicaid program, the research collaboration enabled direct feedback to the Medicaid leadership that can guide efforts to improve care, including outreach to patients. This research suggests that conducting a linkage analysis on an ongoing basis in NYS as well as other states can provide feedback to providers and health systems about performance. This information may assist in targeting efforts to improve care.
